# A New Approach to Supramolecular Structure Determination in Pharmaceutical Preparation of Self-Assembling Peptides: A Case Study of Lanreotide Autogel

**DOI:** 10.3390/pharmaceutics14030681

**Published:** 2022-03-20

**Authors:** Manuela Grimaldi, Angelo Santoro, Michela Buonocore, Claudio Crivaro, Nicola Funicello, Matilde Sublimi Saponetti, Cristina Ripoli, Manuela Rodriquez, Salvatore De Pasquale, Fabrizio Bobba, Lucia Ferrazzano, Walter Cabri, Anna Maria D’Ursi, Antonio Ricci

**Affiliations:** 1 Department of Pharmacy, University of Salerno, Via Giovanni Paolo II, 84084 Salerno, Italy; magrimaldi@unisa.it (M.G.); asantoro@unisa.it (A.S.); mbuonocore@unisa.it (M.B.); mrodriquez@unisa.it (M.R.); 2 Fresenius Kabi iPSUM, Via San Leonardo 23, 45010 Villadose, Italy; claudio.crivaro@fresenius-kabi.com (C.C.); antonio.ricci@fresenius-kabi.com (A.R.); 3 Department of Physics ‘E.R. Caianiello’ of University and Gruppo Collegato INFN, 84084 Salerno, Italy; nfunicello@unisa.it (N.F.); cripoli@unisa.it (C.R.); sdepasquale@unisa.it (S.D.P.); 4 Physics Department and Research Centre for Nanomaterials and Nanotechnology, University of Salerno, Via Giovanni Paolo II, 132, 84084 Salerno, Italy; matildesubli@gmail.com (M.S.S.); fbobba@unisa.it (F.B.); 5 Department of Chemistry “Giacomo Ciamician”, Alma Mater Studiorum, University of Bologna, Via Selmi 2, 40126 Bologna, Italy; lucia.ferrazzano4@unibo.it

**Keywords:** peptides, delivery systems, atomic force microscopy, NMR, MRI

## Abstract

The supramolecular structure in peptides’ prolonged-released gel formulations is the most critical parameter for the determination of the pharmaceutical profile of the drug. Here, we report our investigation on lanreotide Autogel as a case study. For the first time, we describe the use of the pulsed field gradient (PFG) diffusion-ordered spectroscopy (DOSY) magic-angle spinning NMR to characterize the supramolecular self-assembly and molecular mobility of different samples of lanreotide Autogel formulations prepared according to different formulation protocols. The diffusion coefficient was used to calculate the hydrodynamic radii of supramolecular assemblies and build relative molecular models. DOSY data were integrated with NMR imaging (MRI) measurements and atomic force microscopy (AFM) imaging.

## 1. Introduction

Based on the market forecast covering the next 5 years, peptide-based therapeutics are advancing towards having the highest growth with outstanding performances in several diseases such as diabetes, oncology and rare diseases. Indeed, the market is projected to grow from USD 28.5 billion in 2020 to USD 51 billion in 2026, at a CAGR of 9.55% in the forecast period [1].

Chronic diseases often require long-term treatments; therefore, the medicinal chemist’s ability to improve the pharmacological profile of the drug through selective modifications and the increased sophistication and expansion of the drug formulations (nasal, subcutaneous, parenteral and oral) are ever-present challenges, especially in the field of peptide drugs [2,3,4,5,6]. In recent decades, significant efforts have been undertaken in the development of long-acting parenteral formulations (LAPFs) to preserve the drug pharmacokinetics and pharmacodynamics (PK/PD). Several strategies have been developed to design LAPFs, including both in vivo clearance manipulation and drug delivery from delivery systems [7]. The classic chemical modification strategies include PEGylation, PSAylation [8,9,10,11] and lipidation [12,13]. The most innovative and recently developed ones involve fusion with an Fc domain of an immunoglobulin G (IgG) [14,15,16,17] and the use of nanoparticles (NP) [18,19,20]. On the other hand, strategies for manipulating drug delivery from delivery systems include microencapsulation with the formation of microspheres [21,22,23,24], liposomes [25,26,27], oil-based nanoemulsion [28,29,30], nanocrystallization, widely used to improve the properties of soluble drugs [31,32] and the design of hydrogels [33].

One of the methods of designing peptide-controlled release formulations exploits the self-assembly properties of specific amino acid sequences. Some peptide sequences include amino acid sequences that spontaneously generate supramolecular-ordered aggregates. These, through noncovalent intermolecular interactions, maintain the peptide-based self-assembled structures in a stable low-energy state [34,35,36,37,38,39,40]. Self-assembled supramolecular structures, depending on their morphology, exert different physicochemical and biological properties; therefore, they are widely studied as the basis of many neurodegenerative diseases and can be used for intracellular or targeted tissue drug delivery. They are also extensively studied for the prolonged release of peptide drugs, which can deliver monomers in a controlled manner. In this context, the study of proteins and peptide self-association and the parameters that can influence these processes paves the path for a wide range of applications [34,35,36,37,38,39,40].

Many biophysical techniques are used to analyze supramolecular structures: light scattering and optical microscopy, electron microscopy and small-angle X-ray scattering (SAXS), vibrational spectroscopic techniques (Fourier transform infrared and FT-Raman spectroscopies). While most of these analyses require heavy handling of the sample, it is of paramount importance to set an analytical protocol capable of a real-time drug characterization, avoiding sample manipulations. 

**Figure 1:**
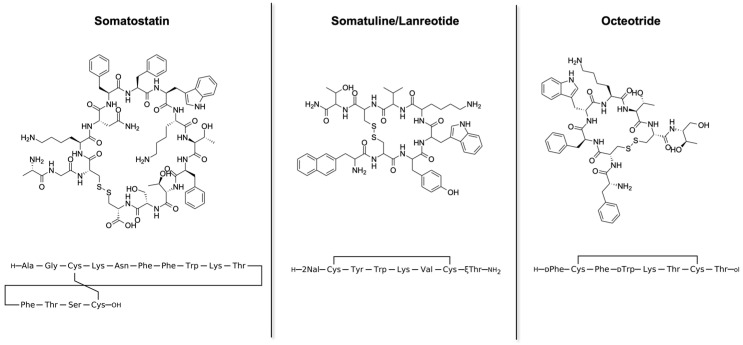
Somatostatin, lanreotide and octreotide peptide structures and sequences.

In this context, lanreotide, a synthetic octapeptide analog of somatostatin (Figure 1), has been extensively studied as a peptide model to investigate protein-like molecular self-assembly processes [41,42]. Lanreotide is currently used for the treatment of acromegaly, grade 1, and a subset of grade 2 gastroenteropancreatic neuroendocrine tumors (GEP-NETs). The drug reduces both growth hormone and insulin-like growth factor-I levels [43,44,45]. In the 1990s, sustained-release (SR) microparticle formulations of two somatotropin release inhibiting factor (SRIF) analogs, lanreotide SR and octreotide long-acting release (LAR), were designed to improve patient compliance, requiring an intramuscular injection every 7–14 days for lanreotide SR and 28 days for octreotide LAR. A recent development approved by the FDA in 2007 was the lanreotide ATG formulation, a supersaturated aqueous solution of lanreotide acetate available in a prefilled syringe, acting as a long-acting formulation extending the dosing interval to 4 weeks with a deep subcutaneous injection [46,47].

Emerging evidence has demonstrated that the counterion is an essential facet of pharmaceutical systems. As excipients in active pharmaceutical ingredients (API), counterions modulate drug efficacy; in peptide formulations, they represent a critical factor to be investigated for their potential to influence peptide lipophilicity, self-assembly tendencies, and formulation stability [48,49]. Furthermore, counterions can be ‘tuned’ to dissociate spatially and temporally to impart desired characteristics: they can be designed to remain ion-paired to the API for a considerable time, even when ionized and dissolved. Such a capability, in turn, modulates drug efficacy, receptor engagement, and cell or nuclear penetration. In addition, the way counterions are added to the formulation is critical to modulate the formulation property [50].

Peptides can accommodate a specific number of counterions according to the acid–basic nature of the amino acids in their primary structure. For self-assembling peptides, the number of counterions is critical to modulate the self-aggregation process and the characteristics of the supramolecular structures. In the case of lanreotide, two basic residues are present (one lysine and the N-terminal amino acid), which allow up to two molecules of acetic acid (AcOH) per lanreotide molecule. The AcOH content is crucial for the lanreotide self-assembly; also, how the counterions are added to the formulation is highly critical [49].

According to the European Patent Application (FDA Somatuline^®^ Leaflet (https://www.accessdata.fda.gov/drugsatfda_docs/label/2014/022074s011lbl.pdf acessed on 27 January 2022)), the preparation of lanreotide ATG in prefilled syringes includes the hydration of API and the homogenization of the final product by recursively transferring the formulation from one syringe to another. AcOH is added during this process to obtain the final optimal amount of counterions.

In the present work, we used NMR spectroscopy and atomic force microscopy (AFM) to analyze samples of lanreotide ATG prepared at different concentrations of AcOH to answer the following questions: (i) In which measure does the acetate amount influence lanreotide ATG self-assembly and the three-dimensional organization of the supramolecular structure? (ii) To what extent does the modality of acetate addition influence the formulation and the stability of a supramolecular structure? (iii) Is it possible to develop an analytical protocol effective for real-time analytical information, avoiding destructive manipulations of the samples and to help us in the understanding of the most suitable method for the preparation of these formulations?

SEM microscopy is the standard analytical method to characterize lanreotide ATG supramolecular self-assembly [51]. However, this technique is, in general, sample-destructive and requires a sophisticated microscopy machine and heavy handling of the sample. Our alternative analytical approach based on the pulsed field gradient diffusion-ordered spectroscopy (PFG-DOSY) and magic-angle spinning (MRI/HR-MAS) NMR spectroscopy allows the characterization of peptide self-assembly in a nondisruptive manner by determining the peptide diffusion coefficient. NMR spectroscopy is integrated with AFM imaging to reconstruct supramolecular folding.

We propose the present protocol as an alternative analytical approach widely applicable to bio-polymer supramolecular structures and useful to guide the development of peptide-based pharmaceutical formulations, used as a reference method in sameness reports.

## 2. Materials and Methods

### 2.1. Sample Preparation

AcOH was purchased from Merck (Rome, Italy) and used without further purification. USP “Water for Injection” grade was used for all the experiments. The lanreotide acetate API powder was produced by Fresenius Kabi (Isola della Scala, Italy) and isolated with different residual AcOH contents for the subsequent formulation experiment. All the samples reported in Table 1 were provided by Fresenius Kabi (Italy) and prepared according to the protocol reported by Ipsen (US9352012B2). Lanreotide acetate API (about 240 mg) was transferred to a 5 mL syringe connected to a second 5 mL syringe through a three-way system.

The second 5 mL syringe was loaded with the appropriate amount of water to reach the concentration of lanreotide acetate available for the US market (Somatuline Autogel^®^, IPSEN Pharma Biotech, Twenty Anson, Singapore), (24.6 mg of lanreotide base per 100 mg of solution) (FDA Somatuline^®^ Leaflet (https://www.accessdata.fda.gov/drugsatfda_docs/label/2014/022074s011lbl.pdf accessed on 27 January 2022)).

**Figure 2:**
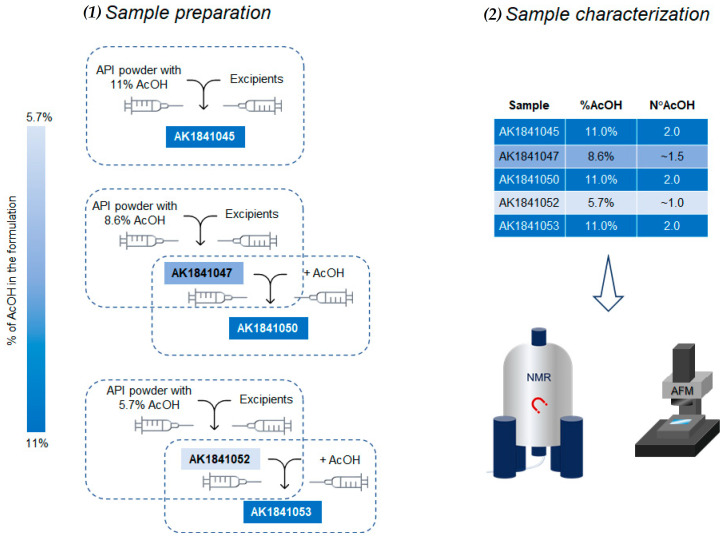
Schematic representation of (**1**) preparation of Somatuline analogs with different amounts of AcOH as indicated by the color bar on the left, and (**2**) the techniques applied for the characterization of supramolecular structures assumed by the obtained formulations.

In the case of AK1841050 and AK1841053 samples, the water used for the reconstitution was added with the amount of AcOH required to reach the final content of 11.0% (corresponding to 2.0 AcOH molecules per lanreotide molecule; see Figure 2).

The water was pushed to the syringe containing the lanreotide acetate, and the obtained mixture was left unstirred for 2 h. The mixture was finally homogenized by pushing the suspension back and forth through the two syringes and finally stored for the subsequent analysis.

### 2.2. HR-MAS Experiments

**Figure 3:**
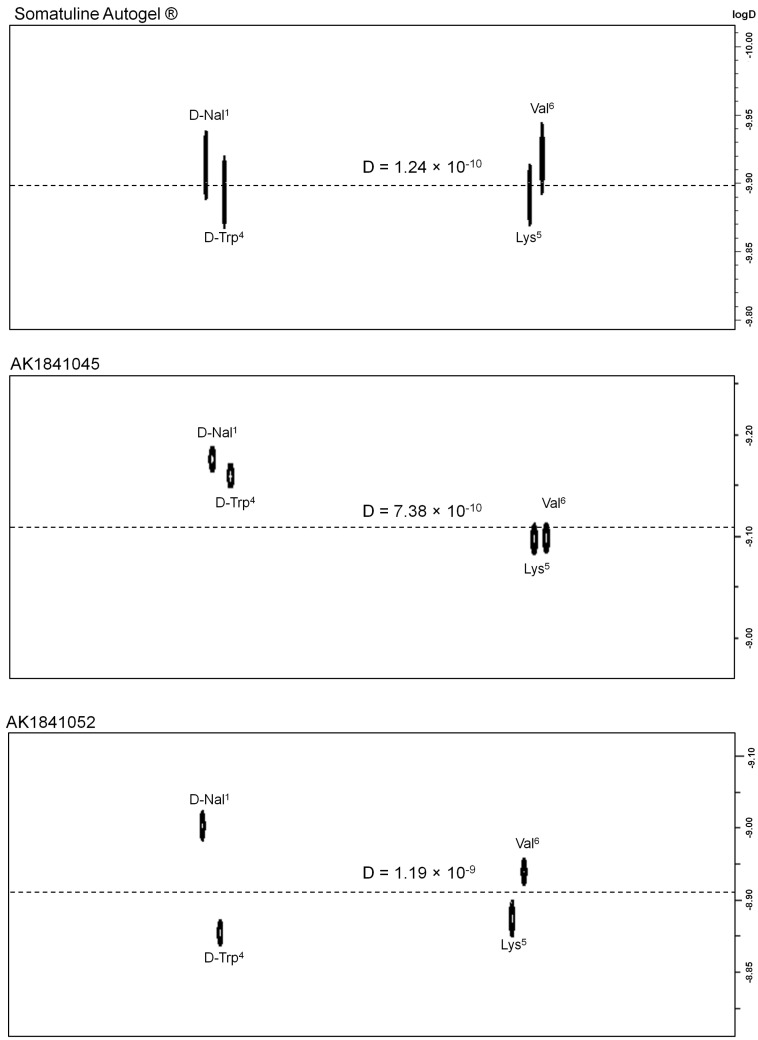
The 2D-DOSY experiments acquired at 300 K on a 500 MHz spectrometer equipped with HR-MAS probe. Spectra reporting the distinctive peaks of Somatuline Autogel^®^, AK1841045 (11% of AcOH), and AK1841052 (5.7% of AcOH), obtained in DMSO-d_6_. The diffusion values were calculated as an average of each peptide peak diffusion value (m^2^/s) and were reported as D values (on the dashed lines) and as logD (ordinate on the right).

All NMR experiments were acquired using Bruker NMR spectrometer 500 MHz equipped with a high-resolution magic-angle spinning (HR-MAS) accessory and a ^1^H/^13^C gradient probe controlled by Topspin 3.2 software package for set-up, acquisition, and processing procedures. The spectra were acquired at a temperature of 300 K, using a 4 mm ZrO_2_ rotor with a hemispherical Teflon insert. For HR-MAS NMR analysis, 40 mg of all the samples was homogenized with 25 μL of DMSO-d_6_; the mixtures were placed inside the rotor and spun at 4 kHz rate with a magic angle of 56°. Pulsed field gradient diffusion-ordered spectroscopy (PFG-DOSY) experiments were acquired (Figure 3). Analysis of DOSY spectra was carried out with Bruker Dynamics Center (Bruker Dynamic Center, Version: 2.6 (26 November 2019). Cys^2^, Tyr^3^, Cys^7^ and Thr^8^ residues were ignored for the D calculation because they were in a crowded area of the spectrum. The average diffusion value (−logD values) was calculated for each sample.

### 2.3. AFM Experiments

Two concentrations were prepared for each sample: 40 μg/μL and 8 μg/μL. The samples were resuspended in water, deposited on mica, and allowed to dry at room temperature. AFM measurements were performed using a JPK NanoWizard^®^ 3 Bioscience system, equipped with a 100 μm scanner, in tapping mode configuration using silicon nitride cantilevers with an elastic constant of 0.6 N/m (MSNL Bruker) and a nominal tip radius of 10 nm. Experiments were performed at a temperature of 22 °C in dry conditions inside a glove box to keep the temperature variation smaller than 0.3 °C/h and to reduce any acoustic noise interferences.

### 2.4. MRI Experiments

Experiments were acquired using Bruker NMR spectrometer 300 MHz/ equipped with Microimaging Probe for Wide Bore Magnets (MicWB40) in combination with the Micro2.5 WB Gradient System. The MicWB40 Probe was developed for microimaging of small objects (max. diam. 30 mm) in wide-bore magnets (89 mm). DOSY experiments were acquired at a temperature of 300 K.

The DWI based on a Spin Echo was the sequence used to produce the diffusion map of the different gels, with one selected direction of diffusion gradients (z-direction). The experiments were acquired at a temperature of 300 K with an acquisition time of approximately 50 min. The parameters used were TR = 3500 ms, TE = 20 ms, matrix size = (128 × 128). The FOV was chosen to cover the whole sample: FOV = 14 mm × 14 mm.

## 3. Results

As previously reported, the lanreotide ATG formulation is a supersaturated aqueous solution of lanreotide acetate. The AcOH concentration is critical for lanreotide aggregation and its long-acting function. However, the way the acetate is added to the lanreotide during formulation has important effects on the structure of the aggregate and, therefore, on the duration of its action.

To understand whether it is necessary to use a full-acetate API powder for an optimal lanreotide ATG formulation or if the addition of AcOH could also occur in multiple steps during the formulation, we performed a combined spectroscopic and atomic microscopy analysis on the lanreotide formulation, where the AcOH content was added to lanreotide during the formulation process. The lanreotide formulations that were the subject of our study are shown in Figure 2, with a schematic representation of how they were obtained. In particular, five formulations of lanreotide acetate, including different AcOH concentrations in the range 5.7–11.0%, were analyzed and compared to Somatuline Autogel^®^ as the reference. First, AK1841045 was obtained by mixing lanreotide API powder containing 11% AcOH (2 AcOH molecules per lanreotide) with an excipient water solution. Next, AK1841047 was obtained by mixing lanreotide API powder containing 8% AcOH (1.5 AcOH per lanreotide molecule) with an excipient water solution; then, this formulation was used to prepare AK1841050 by mixing with AcOH solution to reach an 11% AcOH content. AK1841052 was obtained by mixing lanreotide API powder containing 5.7% AcOH (1 AcOH per lanreotide molecule) with an excipient water solution; then, this formulation was used to prepare AK1841053 by mixing with an AcOH solution to reach an 11% AcOH content.

### 3.1. HR-MAS NMR

The formulations of lanreotide acetate reported in Figure 2 appeared as a highly viscous material that was analyzed by HR-MAS and NMR spectroscopy. Viscous, semisolid samples were characterized by a restricted molecular mobility, resulting in broad NMR lines and not well-resolved NMR spectra. HR-MAS NMR spectroscopy is a relatively new technique in the field of NMR, which allows the obtainment of high-resolution proton spectra of such samples [52,53].

The main advantage of the HR-MAS technique is that it provides direct access to the chemical composition of a sample in its original form, without the need for any laborious sample preparation steps. Thus, qualitative and quantitative spectral information regarding different components in a mixture can be obtained simultaneously.

The size of lanreotide aggregates was determined by acquiring pulsed field gradient diffusion-ordered (PFG-DOSY) experiments. This method allowed us to determine the diffusion coefficient (−logD) of supramolecular aggregates; therefore, their relative size, as described by the Stokes–Einstein equation [54,55]. In Figure 3, we reported the DOSY spectra of Somatuline Autogel^®^ as a reference, and the two formulations with the highest (AK1841045) and lowest (AK1841052) AcOH percentage. The diffusion constants of all the formulations are reported in Table 1.

The analysis of the −logD values indicated that Somatuline Autogel^®^ had the lowest diffusion coefficient (D = 1.24 × 10^−10^; −logD = 9.90). AK1841045, adjusted AK1841050, and AK1841053 formulations, including 11.0% AcOH, showed −logD values comparable to Somatuline, whereas AK1841052 and AK1841047, including lower AcOH percentages, 8.6% and 5.7% AcOH, respectively, showed lower −logD values, consisting of the formation of a less aggregated material (Figure 3).

### 3.2. MRI NMR Experiments

**Figure 4:**
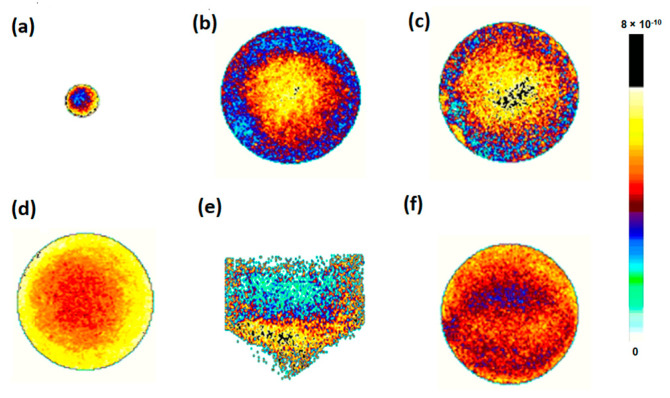
Diffusion experiments acquired using 300 MHz spectrometers equipped with MicWB40 Probe in combination with the Micro2.5 Gradient System. Diffusion maps of axial slices obtained on syringes containing each sample: (**a**) Somatuline Autogel^®^, (**b**) AK1841045, (**c**) AK1841047, (**d**) AK1841050, and (**f**) AK1841053; diffusion maps of the sagittal slice of the syringe containing sample (**e**) AK1841052. The color map on the right indicates D values (m^2^/s): yellowish color indicates a value closer to 8 × 10^−10^, while blueish color indicates a value closer to 0.

Diffusion due to the mobility of water molecules within the samples caused the attenuation of the MR signal through a phase dispersion of the transverse magnetization; diffusion-weighted imaging (DWI) uses magnetic field gradients to detect this dispersion. Figure 4 shows the DWI for each sample. The images show axial slices of the syringe containing the formulations, except for AK1841052, for which it was not possible to obtain an axial slice, only a sagittal one. According to the legend, higher and lower D values (in m^2^/s) were represented in different colors: slower D values were in yellow, and faster D values were in blue. The analysis of the maps indicated the distribution of D values in the sample, providing essential information regarding the homogeneity of the sample. According to the color map and D values obtained (Table 2), the samples Somatuline Autogel^®^ (Figure 4a), AK1841047 (Figure 4c), and AK1841053 (Figure 4f) were those characterized by higher diffusion coefficients; AK1841045 (Figure 4b) and AK1841052 (Figure 4e) had intermediate diffusion values; AK1841050 (Figure 4d) showed lower D values, indicating a more aggregated material.

**Figure 5:**
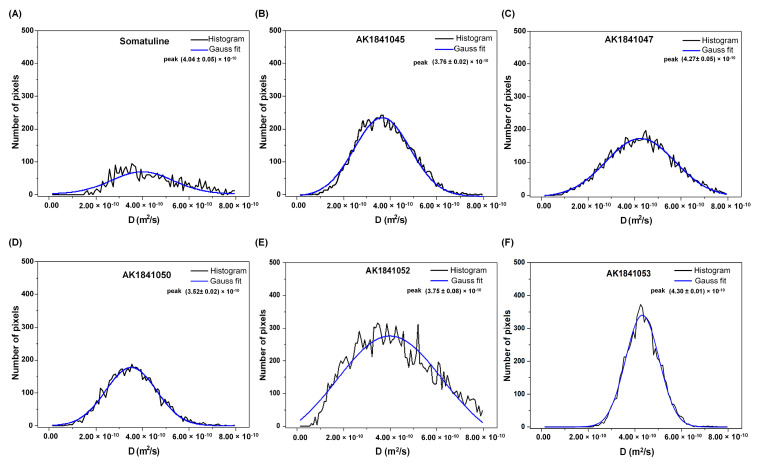
Gaussian distribution of the D values obtained from each pixel in the MRI DWI images of the samples: (**A**) Somatuline Autogel^®^, (**B**) AK1841045, (**C**) AK1841047, (**D**) AK1841050, (**E**) AK1841052, and (**F**) AK1841053. The black lines represent the histogram distributions, and the blue lines report the Gaussian fit. For each sample, the D values are reported, which correspond to the maximum peak of the Gaussian distribution.

Moreover, by looking at DWI images together with the Gaussian distribution of the obtained D values (Figure 5), it was possible to denote an excellent homogeneity in samples AK1841045 (Figure 5B), AK1841050 (Figure 5D), and AK1841053 (Figure 5F), and a rather good homogeneity in sample AK1841052 (Figure 5E). Therefore, MRI was a valuable and immediate tool in analyzing the formulation’s consistency.

### 3.3. AFM Microscopy

**Figure 6:**
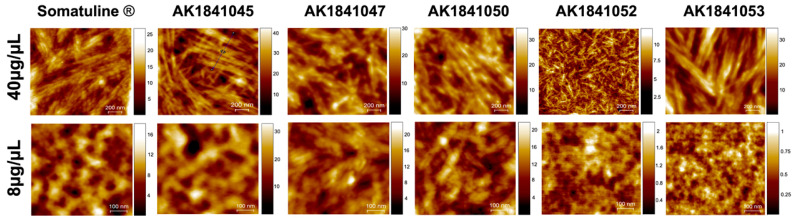
Topographic AFM images (0.6 × 0.6 µm^2^) acquired on mica at room temperature in dry conditions. For each sample, we acquired two concentrations (40 μg/μL and 8 μg/μL). AFM measurements were performed in tapping mode configuration using silicon nitride cantilevers with an elastic constant of 0.6 N/m (MSNL Bruker) and a nominal tip radius of 10 nm.

Atomic force microscopy is a powerful technique that, with a resolution of nanometers, allows the characterization of the structure and function of minuscule and soft samples, such as cells, proteins, and DNA, in physiological conditions [56]. Many parameters must be considered to accurately image biological samples, such as optimizing their fixation on the substrate, selecting the proper cantilever and imaging mode, and applying low-interaction forces. In this regard, in our study, we characterized the size of the lanreotide formulations using the tapping imaging mode. The parameters considered as relevant were the height distribution after the surface modification and the shape preservation. Figure 6 shows the topographic AFM images of Somatuline Autogel^®^ and its analogs. In particular, AK1841047 and AK1841052 (D = 2.49 × 10^−9^ m^2^/s and D = 1.19 × 10^−9^ m^2^/s, respectively) were characterized by short, low, and tightly packed tubular structures. AK1841053 and AK1841050 ribbon macrostructures seemed to be present, but their self-organization in the cross-linked structure was not discernible.

**Figure 7:**
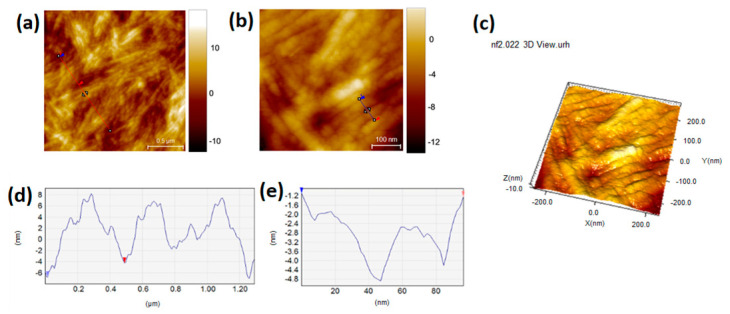
Topographic AFM images (1.5 × 1.5 μm^2^ (**a**), 0.6 × 0.6 μm^2^ (**b**) and 3D (**c**)) of Somatuline Autogel^®^ (**d**,**e**). Graphs showing the height line profiles measured on the topographies indicated by the blue lines. AFM images show the presence of tubular structures that self-organized in ribbons. Graphs display the height and width of ribbons (**d**) and tubular structures (**e**). AFM measurements were performed in tapping mode on dry samples.

**Figure 8:**
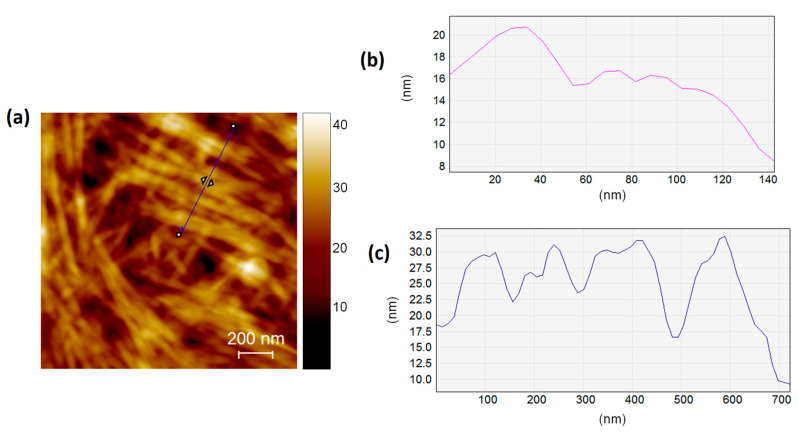
AFM height image (1.5 × 1.5 μm^2^) of AK1841045 (**a**) and height line profile measured on the topographies indicated by blue and pink lines (**b**,**c**). AFM images show the presence of tubular structures which self-organized in ribbons. Graphs display the height and width of tubular structures (**b**) and ribbons (**c**). AFM measurements were performed in tapping mode on dry samples.

**Figure 9:**
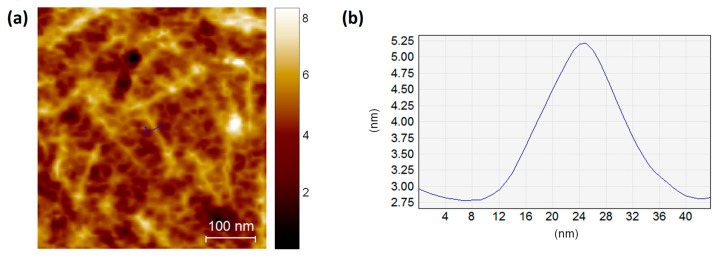
AFM height image (0.6 × 0.6 μm^2^) of AK1841052 (**a**) and height line profile (**b**) measured on the topography indicated by the blue line. AFM measurements were performed in tapping mode on dry samples.

The height line AFM profiles were also shown for Somatuline, AK1841045, and AK1841052 (Figure 7, Figure 8 and Figure 9). The topographic AFM image and height line profile of Somatuline Autogel^®^ (Figure 6) showed that large cross-linked tubular structures tended to self-organize in ribbons, ranging from 80 to 500 nm in diameter. In particular, the height line profiles showed ribbons with a diameter of 200–500 nm and tubular structures with diameters of 30–40 nm.

The height line profile of AK1841045 (Figure 8) indicates that this sample was characterized by tubular structures with about 20 nm in diameter and ribbons with an 80–200 nm diameter (Figure 8b,c).

In particular, AK1841052 presented almost disaggregated tubes with an average diameter of 20 nm, as shown in the height line profile (Figure 9b).

## 4. Discussion

To date, dozens of peptide-based drugs have been approved by the FDA to treat chronic diseases. Drugs for chronic diseases require the development of long-acting formulations that allow for a reduced number of administrations and, therefore, improve patient compliance.

Lanreotide, a synthetic octapeptide analog of somatostatin, is currently used for the treatment of acromegaly. It is available as a long-acting formulation consisting of a supersaturated aqueous solution of lanreotide acetate. The production of lanreotide ATG exploits the intrinsic attitude of lanreotide to form self-assembling supramolecular structures. Peptide self-assembly depends on several conditions: the peptide environment (solvent), temperature, concentration, pH, enzymes, and co-solved molecules, but mainly on the nature and content of counterions [46,47,48]. Indeed, counterions, acting as kosmotropic or chaotropic agents, may be critical for the aggregation of kinetic or aggregate stability and, as such, [48] have a decisive influence on peptide drug formulation in pharmacokinetics and stability properties.

The lanreotide ATG formulation critically depends on the appropriate content of AcOH counterions. In the present work, we investigated: (i) how AcOH counterions condition the ability to form supramolecular structures in lanreotide formulations; (ii) we exploited an alternative, innovative analytical approach based on NMR spectroscopy and AFM microscopy to improve the performance of the standard analytical procedures. 

The standard methodologies used to study self-assembled peptide-based formulations are size-exclusion chromatography [57], ultracentrifugation [58], FFF [59], SEM [51] and small-angle X-rays [60]. However, these techniques generally require the complete dissolution of the sample and/or impose a perturbation of the peptide aggregate, jeopardizing the native properties of the formulation [61].

The analysis of DOSY spectra in the semisolid state (HR-MAS) showed that the diffusion rates and the tendency of the peptide to self-assemble vary with the content of AcOH (5.7% AK1841052 and 8.6% AK1841047). Therefore, lanreotide formulations including a high AcOH content tended to form complex supramolecular structures. Integrating these findings, MRI NMR data provided information on the texture and homogeneity of the samples, features that are critical for a high-quality formulate preparation. It is worth noting that the NMR MRI analysis did not require any manipulation of the samples, including the sample transfer from the prefilled syringe to a different NMR tube. The data collected with NMR DWI showed the diffusion coefficient of the water molecules in the samples as distributed on a Gaussian curve. Accordingly, an excellent material homogeneity was evident in the samples with the highest percentages of acetate (AK1841045, AK1841050 and AK1841053); on the contrary, less homogeneous samples were those with a low acetate content, such as AK1841052.

Finally, the AFM technique provided valuable information regarding the organization of the supramolecular structures. Somatuline Autogel^®^, as well as AK1841045 (11.0% AcOH), exhibited large cross-linked tubular structures with the tendency to self-organize in ribbons with different diameter tubular structures. AK1841053 and AK1841050 formulations, with the adjusted percentage of AcOH, showed ribbon macrostructures, but self-organization was not discernible. AK1841047 and AK1841052 formulations, with the lowest percentage of AcOH, were characterized by short, low, and tightly packed tubular structures. All these data strengthened the crucial role of the AcOH counterion in the formation of the supramolecular structure. In particular, the AcOH pairing of both of the two NH_2_ Lysine side chains and terminal backbone NH_2_ was an essential requisite for a supramolecular organization similar to the Somatuline Autogel^®^.

## 5. Conclusions

The development of an analytical methodology to monitor the preparation of drug formulations with a negligible perturbation of the sample is a hot area of investigation. In fact, this has a huge impact on guiding the formulation development and guaranteeing a batch-to-batch comparison in sameness protocols.

In the present work, we described the characterization of lanreotide acetate using a combination of imaging and magic-angle spinning NMR spectroscopy with atomic force microscopy. Moreover, we demonstrated the potentiality of counterions, such as AcOH, to influence the auto-assembly process with the formation of specific supramolecular structures. All our data suggest that the formulations containing the highest percentage of acetate were characterized by large cross-linked tubular structures with the tendency to self-organize in ribbons, such as for AK1841045 (11% AcOH). Adjusting the percentage of AcOH, such as for the AK1841053 and AK1841050 formulations, ribbon macrostructures were observed, but self-organization was not discernible. Therefore, the addition of AcOH during the formulation process prevented the formation of optimal supramolecular structures; thus, proving that two molecules of AcOH bound at the stage of the lyophilized powder were necessary for a complete organization of the lanreotide tridimensional structure.

Our studies on lanreotide Autogel formulations showed that the characterization of complex macromolecular structures is possible by measuring diffusion coefficients based on DOSY NMR experiments. These methods are fast, economical, and conservative compared to SEM, which is considered the reference technique for these characterizations. Moreover, this study proposed MRI NMR as a promising technique to characterize the formulation’s material texture. These analytical protocols proved to be suitable in guiding the formulation development and represent a powerful technique in showing peptide formulation sameness in the case of gel formulations.

## Figures and Tables

**Table 1:** Diffusion (D) values (m^2^/s) and the respective −logD transformation calculated from HR-MAS DOSY spectra on each sample.

Sample	%AcOH	HRMAS D Values (m^2^/s)	−logD Values
Somatuline Autogel^®^		1.24 × 10^−10^	9.90
AK1841045	11.0%	7.38 × 10^−10^	9.13
AK1841047	8.6%	2.49 × 10^−9^	8.60
AK1841050	Adjusted to 11.0%	7.67 × 10^−10^	9.11
AK1841052	5.7%	1.19 × 10^−9^	8.92
AK1841053	Adjusted to 11.0%	7.69 × 10^−10^	9.11

**Table 2:** Diffusion (D) values (m^2^/s) and the respective −logD transformation calculated from MRI DWI on each sample.

Sample	%AcOH	MRI D Values (m^2^/s)	−logD Values
Somatuline Autogel^®^	11.5%	(4.04 ± 0.05) × 10^−10^	9.39
AK1841045	11.0%	(3.76 ± 0.02) × 10^−10^	9.42
AK1841047	8.6%	(4.27 ± 0.05) × 10^−10^	9.37
AK1841050	Adjusted to 11.0%	(3.52 ± 0.02) × 10^−10^	9.45
AK1841052	5.7%	(3.75 ± 0.08) × 10^−10^	9.42
AK1841053	Adjusted to 11.0%	(4.30 ± 0.01) × 10^−10^	9.36
